# Supplementary material for: Modelling the ability of mass drug administration to interrupt soil-transmitted helminth transmission: Community-based deworming in Kenya as a case study
Source: PLoS Negl Trop Dis. 2021 Aug 2;15(8):e0009625. doi: 10.1371/journal.pntd.0009625 (PMC8360579; doi:10.1371/journal.pntd.0009625)
Supplement: Attachment — Submitted filename: ResponsesToReviewersComments.pdf [file pntd.0009625.s001.pdf]

# Modelling the ability of mass drug administration to interrupt soil-transmitted helminth transmission: community-based deworming in Kenya as a case study

Dear Dr. Guo-Jing Yang and Dr. Banchob Sripa,

Thank you for the reviews of our manuscript, which were extremely helpful. We have reworked and rewritten the manuscript in light of the comments. We have done everything the reviewers requested. Here is a point-by-point response to the reviewers.

## Reviewer # 1

This reviewer had in total 26 comments.

**Response**: We have done almost everything this reviewer requested. Changes due to this reviewer are in blue.

**Comment (1):** I am unable to make any comment about the mathematical modelling, but if you are treating worms and of equal importance to public health across species it is flawed as 100 eggs of hookworm cause more morbidity than 100 of T. Trichuria v 100 of Acscaris.

**Response:** This is true, but we are not modelling worms by species, but instead aggregating in a general sense.

**Comment (2):** Application to the Tumikia project in Kenya.
L 269: Is this project aiming to control or to eliminate?

**Response:** This project aims for both control and elimination [4]. We have added a note to this effect. (Page 14, lines 311–313)

**Comment (3):** L 274: This sounds as if after 2 years you expect the problem to have been solved without potential recrudescence. Within 2 years you may get

reduction or even control ($< 10\%$ prevalence with $< 1\%$ moderate/heavy infections) but recrudescence is inevitable of behaviours and WASH resources are not addressed no matter how many rounds of effective drugs are used.

**Response:** We thank reviewer for flagging this point. We have added a clearer objective in this section. (Page 15, lines 325–326)

**Comment (4):** L 335: You do not take into consideration that STH eggs remain viable in soil for many years, hence this interruption is only temporary.

**Response:** This is a good point and an important limitation to our findings. We have mentioned this in the Discussion. (Page 19, lines 391–392)

**Comment (5):** L 341 Eradication is a completely new term and not appropriate here. Even elimination is inappropriate. I believe you are aiming for control whilst, WASH and human behavioral changes are strengthened.

**Response:** We have changed the wording. (Page 19, line 386)

**Comment (6):** L 2: I suggest 'control' rather than 'tackle'.

**Response:** We have changed to "control" as suggested. (Page 1, line 2)

**Comment (7):** L 3: Can you make any comment regarding the cost of using an additional round of weaker drugs vs cost of using stronger drugs and fewer rounds?

**Response:** Unfortunately, we do not have any data about this question at the moment; however, we have provide some information regarding cost of STH treatment in children vs adults (Page 2, lines 48–57)

**Comment (8):** Abstract.
Responsive to 'preventive chemotherapy'.
Is the mean number of worms the best way of perceiving the effort to reduce STH to "no longer of public health significant" ($< 10\%$ prevalence of any STH and $< 1\%$ moderate/heavy infections) Do you actually mean worms of eggs per gram of faces? There is such enormous variance in the morbidity of epg by species that you cannot compare 100epg for hookworm with 100epg for Ascaris: the morbidity is grossly different.

**Response:** No, the mean number of worms in this article does not refer to worms of eggs per gram of faeces. The mean number of worms ($M$) in this article refers to the average number of worms in a human population of density $N$ at time $t$. We have made our definition explicit. (Page 4, lines 115–116, in red, as the other reviewer also raised this point.)

**Comment (9):** L 12: I suggest 'preventive chemotherapy (PC)' is more appropriate than 'deworming' as one doesn't know if the individual has actually been de-wormed the worm burden will have reduced but whether it gets to zero will depend upon mostly the worm burden before treatment as well as the type of medicine used (more v less effective).

**Response:** Thank you very much for the suggestion and we have changed the "deworming" to "preventive chemotherapy" throughout. (Page 2, line 9 etc.)

**Comment (10):** L 18: I don't think 'only' is helpful here as targeted chemo and selective chemo and mass chemo often coexist in a community.

**Response:** We thank reviewer for flagging this point. We have removed the word "only". (Page 2, line 18)

**Comment (11):** L 19: I don't think you need 'after a regular screening test'. Persons can self-refer for selective chemotherapy and be treated by clinicians/pharmacists without tests and/or buy over the counter medicines without consultations.

**Response:** We have done this and thank the reviewer for the suggestion. (Page 2, line 19)

**Comment (12):** L 21: Of doses of PC rather than 'treatments'.

**Response:** Done. (Page 2, line 20)

**Comment (13):** L 25: This brings be back to my comment about the costs of providing another round of MDA with a less effective agent versus the cost of less rounds of MDA with more effective agents.

**Response:** We would like to apologize for unable to make any comment regarding this question as we do not have any data/information about this question. However, we provide some information; please refer to Comment (7).

**Comment (14):** L 26: PC should have been introduced earlier and then the abbreviation can be used throughout the manuscript.

**Response:** Thanks. We have fixed it throughout this manuscript.

**Comment (15):** L 30: I would add that the strategy needs to be respectful of the local context, traditional authorities, customs and belief systems if the last mile toward STH control is to be effective and that control to be maintained. I suggest that STH recrudesce is almost inevitable if these are not taken into consideration: if

personal and environment hygiene are not considered.

**Response:** This is an excellent point. We have done added this. (Page 2, lines 25–26 and Page 19, lines 394–395)

**Comment (16):** L 33: This is a different definition to that used by the WHO 'no-longer of public health significance'.

**Response:** We have fixed this/ (Page 2, lines 31–33)

**Comment (17):** L 34: Important to also mention other important control strategies: improved water and sanitation (WASH).

**Response:** Good point. We have done so. (Page 2, line 34)

**Comment (18):** L 38: Or community-based.
**Response:** We have done this. (Page 2, line 38)

**Comment (19):** L 41: Rather than use the word 'treating' I would use PC.

**Response:** We have changed this. (Page 2, line 43)

**Comment (20):** L 44: Trichuris trichiura. The combination of ALB and IVM is recommended for LF-PC.

**Response:** We have changed this. (Page 2, lines 46–47)

**Comment (21):** L 45: T. trichiura.

**Response:** Thanks. We have fixed this. (Page 2, line 47)

**Comment (22):** L 48: Only strategy? No health education on WASH and/or efforts to improve safe water and improved sanitation?

**Response:** We have improved this comment. (Page 2, lines 63–65)

**Comment (23):** L 53: Did this study continue to validate how long before STH recrudesce occurred if there truly was no strategy for WASH?

**Response:** No, we did not include this. We have added this to our study limitations (Page 19, lines 393–394).

**Comment (24):** L 63: Areas with high baseline prevalence are especially in need of health education on personal hygiene and improved sanitation at household, community and school-level. MDA alone will be insufficient to eliminate and prevent

resurgence. Rebound STH infection after mass MDA and apparent ‘control’ has been well documented for over 30 years.

**Response:** Good point, and we thank reviewer for this comment. (Page 3, lines 79–82)

**Comment (25):** L 73: Or consistently don’t or cannot adopt improved WASH practices.

**Response:** We have added this comment. (Page 3, lines 92–93)

**Comment (26):** L 76: Again you could consider the cost-benefit of this approach, identifying monitoring and treating groups of individual versus improving WASH resources for a community/ or a vulnerable subgroup within a community.

**Response:** We thank the reviewer for the good point. (Page 3, lines 93–99)

## Reviewer # 2

This reviewer had 23 comments.

**Response**: We have done everything this reviewer requested. Changes due to this reviewer are in red.

**Comment (1):** What is the mean number of worms in a population? The mean of the estimate? The number of worms in a population is a scalar, not a distribution.

**Response:** We have clarified this. (Page 4, lines 115–116 and line 119)

**Comment (2):** Figure 1 shows the mean value of worms for different R0. There are three lines to correspond to three values of k, without any explanation as to why these values were chosen. The relation of k to the real life is lacking, i.e. it is impossible to interpret what different "clumping parameters of the negative binomial distribution" mean in the real world. What different settings would have a low/high k?

**Response:** We thank reviewer for the good points. We have added in an explanation for our choices of $k$. (Page 5, lines 134–139)

**Comment (3):** Why provide the eigenvalues of the models? What does the eigenvalue tell us?
Lines 116, "Moreover, the endemic equilibrium of model (2) is locally asymptotically stable whenever [an eqn where any relationships between parameters are too complicated to make the eqn easily interpretable] since all associated parameters are positive." What real world information does this tell us about the endemic equilibrium? Similarly on line 216.

**Response:** This raises a good point. Eigenvalues are used to determine stability of equilibria for multidimensional systems. However, since our system is one-dimensional, the eigenvalue is equivalent to the derivative. We have thus streamlined our argument and added an explanation of why we are interested in stability of equilibria. This has simplified the complexity and hopefully not alienated readers for whom linear algebra may be a distant memory! (Page 5, lines 140–147 and Page 6, 154–155)

**Comment (4):** L 177 "To find the endpoints of an impulsive orbit..." What is an impulsive orbit? Why do we need to calculate them?

**Response:** We have added an explanation. (Page 7, lines 163–168)

**Comment (5):** There is regular mention of choosing arbitrary parameter values. Why? Are real life values impossible to obtain? In which case, why? Because they don't relate to real life or because the data is difficult to obtain?

**Response:** Yes, some of the real parameter values are difficult to obtain, such as the clumping parameter ($k$), $R_0$ and initial values especially in specific region, etc. In these cases, we choose arbitrary parameter values that fulfill certain conditions if there are any (for example, the conditions as stated in the claim of Comment (3)).

**Comment (6):** How is the drug efficacy interpreted? Is it assuming perfect adherence? Is it the clearance rate?

**Response:** We have added an explanation. (Page 7, lines 194–195)

**Comment (7):** With regards to writing style - it is unclear what parts of the model are new and the authors contribution to the field.

**Response:** All models in this paper are new except models (1) and (2). Models (1) and (2), proposed by Anderson and May [1], are our inspiration for the study, but we develop other models, such as (3)–(4) and (8). We have made the source explicit. (Page 4, lines 117–118) We have also added a formal section for authors' contributions. (Page 19, lines 402–406)

**Comment (8):** The application to Kenya data is underwhelming. The prevalence is averaged over the whole country, making the application very broad. It is not shown methodically that only (b) and (c) strategies are considered in this paper. There is a lack of clarity with regards to how many MDA rounds are used in (b) compared to (c). Results are converted from decimals to percentages in an inconsistent manner. The plots are provided without explanation as to the meaning of $\hat{M}$ etc (plots should be intepretable without having to read the paper).

**Response:** We have been guided by the impact of TUMIKIA community-based biannual and annual deworming strategies. We have added the number of rounds of MDA (Page 15, lines 323–324), converted all results to decimal points and fixed all the plots. (Figures 2–12) We thank the reviewer for flagging these.

**Comment (9):** Abstract
- Don't include parameter notation in the abstract.
- The acronym STH is introduced early on, and then throughout the paper the authors switch from writing out soil-transmitted helminthiasis in full, and using STH.

**Response:** We have fixed the former. (Page 1) For the latter, you are correct, but we have been careful to use "STH" for "soil-transmitted helminth" while writing out "soil-transmitted heminthiasis" in full.

**Comment (10):** Line 38. The idea of focusing on children is alluded to, but not formally addressed.

**Response:** We have done this. (Page 2, lines 38–40)

**Comment (11):** Line 52 How low for R0?

**Response:** We have done this. (Page 3, line 63)

**Comment (12):** Line 53 Did Clark et al. [10] use data? This is unclear.

**Response:** We have added a note to clarify the methodology of Clark *et al.* [10] (Page 3, lines 68–69)

**Comment (13):** Line 64 Is MDA the deworming strategy?

**Response:** Yes. We have made this explicit. (Page 2, line 14)

**Comment (14):** Line 68 states that there is variation in R0. In what sense? Under different settings? This statement doesn't provide enough information to make the reader feel that the work had a conclusion.

**Response:** We have fixed this. Thanks. (Page 3, lines 85–87)

**Comment (15):** Line 81 to 86 Use the same wording where possible, so that the differences between the two models are immediately clear. Similarly for lines 275-280.

**Response:** We have made these more clear. (Page 3, lines 104–108, and Pages 14–15, lines 319–325)

**Comment (16):** Line 87-91 and line 212 are missing references to Sections.

**Response:** We thank the reviewer for pointing these out. We have revised these. (Page 4, lines 109–113 and Page 11, line 257)

**Comment (17):** Line 97 needs a reference to justify using a negative binomial distn.

**Response:** We have added a reference. (Page 4, line 119)

**Comment (18):** Table 1 would benefit from z being added, even if stating $e^{-\gamma}$, to make the introduction to the model smoother.

**Response:** Good suggestion. Done. (Page 4, Table 1.)

**Comment (19):** The notation $M_{eq}, M^*, M_*$ is unclear, and I'm not sure whether the authors interchange parts of these. In general, $M^*$ and $M_*$ are not distinctly different enough.

**Response:** In general, $M_{eq}$ is denoted as endemic equilibrium of the model in this manuscript. Since we obtain more than one endemic equilibrium, we would like to categorize them into a stable endemic equilibrium, which is denoted as $M^*$, and an unstable endemic equilibrium, which is denoted as $M_*$. This is standard notation in the mathematical literature, so we prefer to keep it.

**Comment (20):** Line 125, n is a positive arbitrary integer. What are the units?

**Response:** There is no unit for this $n$ as it is a notation to differentiate the time $t$ (integers are unitless). However, the unit of time $t$ in our study is year.

**Comment (21):** Line 155 states that if k is large. From Fig 1 can you relate this to R0, and instead state that when R0 is large. This is a better reference point because R0 has real life meaning.

**Response:** We have done this. (Page 7, lines 200–201)

**Comment (22):** Line 176 What are these functions!?

**Response:** $g_1(t_i)$ and $g_2(t_i)$ are any input functions whose mean squared error we would like to find. For instance, to find MSE as in Figures 4 and 5, $g_1(t_i)$ could represent the solution of model (3)–(4), whereas $g_2(t_i)$ represents the analytical solution (5) with (6), and vice-versa. However, to find MSE as in Figures 8 and 9, $g_1(t_i)$ could represent the solution of model (12)–(13), whereas $g_2(t_i)$ represents the solution of model (9)–(10), and vice-versa. We have tweaked this to make it clear that we are talking generally. (Page 9, Line 221)

**Comment (23):** For a public health journal, like the abstract, the discussion should avoid use of referring to parameters with their notation (i.e., k).

**Response:** Good point. We have described the parameters in words. (Page 18, line 377)

**Reviewer # 3**

This reviewer notes that "The problem that this manuscript takes in consideration is of great importance in some regions of the world. The authors carry on their analises with the help of some Mathematical models described by ODEs systems. Of special interest is the carefulness that the author put in the derivation of the form of constitutive functions. The numerical results are carefully compared with real data. The author discuss about some possible restrictions on the applicability of their study.The paper is clear and well organized, in my opinion it can be published in the present form." There is no change to be made.

In summary, we feel that these revisions have addressed all the points raised by the reviewers and hope that the manuscript is now acceptable.

Yours sincerely,

Nyuk Sian Chong, Stacey Smith?, Marleen Werkman and Roy Anderson

# References


[1] Anderson, R. M. & May, R. M. 1991. *Infectious Diseases of Humans: Dynamics and Control.* 1st ed. Oxford: Oxford University Press.

[2] Anderson, R. M., Truscott, J. E. & Hollingsworth, T. D. 2014. The coverage and frequency of mass drug administration required to eliminate persistent transmission of soil-transmitted helminths. *Phil. Trans. R. Soc. B* 369: 20130435.

[3] Anderson, R. M., Turner, H. C., Truscott, J. E., Hollingsworth, T. D. & Brooker, S. J. 2015. Should the goal for the treatment of soil transmitted helminth (STH) infections be changed from morbidity control in children to community-wide transmission elimination? *PLoS Negl Trop Dis*, 9(8): e0003897.

[4] London Centre for Neglected Tropical Disease Research. TUMIKIA: evaluation of school- versus community-based deworming on the transmission of STH in coastal Kenya.
`https://www.londonntd.org/research/projects-search/tumikia-evaluation-of-school-versus-community-based-deworming-on-the`
Accessed March 25, 2021

[5] Mascarini-Serra, L. 2011. Prevention of soil-transmitted helminth infection. *J Glob Infect Dis*, 3(2): 175–182.

[6] Midzi, N., Montresor, A., Mutsaka-Makuvaza, M. J., Fronterre, C., Manangazira, P., Phiri, I., et al. 2020. Elimination of STH morbidity in Zimbabwe: Results of 6 years of deworming intervention for school-age children. *PLoS Negl. Trop. Dis.*, 14(10): e0008739.

[7] Perko, L. 2001. *Differential equations and dynamical systems.* 3$^{\text{rd}}$ ed. Springer, New York.

[8] Truscott, J. E., Hollingsworth, T. D., Brooker, S. J. & Anderson, R. M. 2014. Can chemotherapy alone eliminate the transmission of soil transmitted helminths? *Parasites & Vectors*, 7: 266.

[9] Wiggins, S. 1990. *Introduction to applied nonlinear dynamical systems and chaos.* Springer, New York.

[10] World Health Organization. 2021. Soil-transmitted helminth infections. `https://www.who.int/news-room/fact-sheets/detail/soil-transmitted-helminth-infections` Accessed March 25, 2021

[11] Albert Einstein. *Zur Elektrodynamik bewegter Körper.* (German) [*On the electrodynamics of moving bodies*]. Annalen der Physik, 322(10):891921, 1905.

[12] Knuth: Computers and Typesetting, `http://www-cs-faculty.stanford.edu/~uno/abcde.html`
