# Supplementary material for: Modelling the ability of mass drug administration to interrupt soil-transmitted helminth transmission: Community-based deworming in Kenya as a case study
Source: PLoS Negl Trop Dis. 2021 Aug 2;15(8):e0009625. doi: 10.1371/journal.pntd.0009625 (PMC8360579; doi:10.1371/journal.pntd.0009625)
Supplement: Attachment — Submitted filename: ResponsesToReviewersComments.pdf [file pntd.0009625.s002.pdf]

Modelling the ability of mass drug administration to interrupt soil-transmitted helminth transmission: community-based deworming in Kenya as a case study

Dear Dr. Guo-Jing Yang and Dr. Banchob Sripa,

Thank you for the reviews of our manuscript, which were extremely helpful. We have reworked and rewritten the manuscript in light of the comments. We have done everything the reviewers requested. Here is a point-by-point response to the reviewers.

Reviewer # 1

This reviewer noted that the manuscript is much improved and now fits better into the reality of programming in SSA. This reviewer had 18 minor comments.

Response: We have done everything this reviewer requested. Changes due to this reviewer are in blue.

Comment (1): L392: ‘so human-induced interruptions are only temporary; hence our results have less viability in the long term’ could you phrase this. Perhaps something like ‘recrudescence of STH is inevitable without improvements in access to WASH and the adoption of new behaviors by the communities affected’.

Response: Thanks, this is a great suggestion. We have done so. (Page 19, lines 393–395)

Comment (2): L394: and SBCC

Response: Added. (Page 19, line 396)

Comment (3): L 396-398: could you also include a cost-benefit analysis of these various schedules of MDA. Implementing MDAs takes time, coordination and costs. Volunteers are increasingly expecting to be paid for their services so a more effective

drugs delivered less frequently might be significantly cheaper for a health service than a cheaper drug requiring more rounds of MDA.

Response: We really like this suggestion! We've added it and are very excited to take this on. (Page 19, line 401)

Comment (4): L22: 'deworming' use instead of 'PC' or MDA which is used more often going forward

Response: We've standardised this. (Page 2, line 22)

Comment (5): L24-27: you have omitted the human-element: the change in behavior usually addressed by Social and Behavior Change Communication (SBCC)

Response: Excellent addition, thanks. (Page 2, lines 27–28)

Comment (6): L29: you have used PC here but almost everywhere else you have used MDA, please be consistent unless you are trying to differentiate between MDA and PC?

Response: We have changed this to MDA. (Page 2, line 29)

Comment (7): L31: 'deworming'

Response: Changed to MDA. (Page 2, line 31)

Comment (8): L34: and SBCC .

Response: Added. (Page 2, line 34)

Comment (9): L39: now MDA is used instead of PC, please choose on or the other but don't switch back and forth

Response: We've kept MDA.

Comment (10): L47: Trichuris trichiura has been introduced in full on L46 so it can be abbreviated to T. trichiura form then onwards

Response: Oh yes, good catch. (Page 2, line 47)

Comment (11): L48-49: Schools are not targeted because of cost-efficiencies but because the SAC are, in untreated communities the carriers of the highest burden of STHs and whilst growing suffer the greatest set-backs to growth, health and cognition.

Response: These are excellent observations, thanks. (Page 2, lines 48–51)

Comment (12): L56: and BCCC (which is different from just health education)

Response: We assume this is a typo for SBCC, which we've added. (Page 3, line 58)

Comment (13): L58: MDA (or PC) but not 'deworming'

Response: Fixed. (Page 3, line 60)

Comment (14): L89: 'deworming'

Response: Changed to MDA. (Page 3, line 90)

Comment (15): L98: presumable also of SBBC?

Response: They don't use that term, but they do talk about behaviour change, so we've added that. (Page 3, line 99)

Comment (16): L373: 'appropriate' what is meant by that?

Response: We changed it to "sufficient" and have also rewritten the sentence. See next point. (Page 18, line 376)

Comment (17): L375: 'greater than' or less than?

Response: It is actually greater than (if it was less than the breakpoint, the control strategies would not be needed), but we agree that this was confusing, so we have reworded to make this clear. (Page 18, lines 374–376)

Comment (18): L375 'Otherwise, the infection may persist' This sounds odd do you mean the infection in that individual or the risk of recrudescence within the community?

Response: The latter. We've clarified. (Page 18, line 377)

In summary, we feel that these revisions have addressed all the points raised by the reviewers and hope that the manuscript is now acceptable.

Yours sincerely,
